# Expression and contributions of the Kir2.1 inward-rectifier K^+^ channel to proliferation, migration and chemotaxis of microglia in unstimulated and anti-inflammatory states

**DOI:** 10.3389/fncel.2015.00185

**Published:** 2015-05-12

**Authors:** Doris Lam, Lyanne C. Schlichter

**Affiliations:** ^1^Genetics and Development Division, Toronto Western Research Institute, University Health NetworkToronto, ON, Canada; ^2^Department of Physiology, University of TorontoToronto, ON, Canada

**Keywords:** *KCNJ2*, ML133, alternative-activated microglia, acquired-deactivated microglia, interleukin-4 stimulation, interleukin-10 stimulation, microglial migration, microglial proliferation

## Abstract

When microglia respond to CNS damage, they can range from pro-inflammatory (classical, M1) to anti-inflammatory, alternative (M2) and acquired deactivation states. It is important to determine how microglial functions are affected by these activation states, and to identify molecules that regulate their behavior. Microglial proliferation and migration are crucial during development and following damage in the adult, and both functions are Ca^2+^-dependent. In many cell types, the membrane potential and driving force for Ca^2+^ influx are regulated by inward-rectifier K^+^ channels, including Kir2.1, which is prevalent in microglia. However, it is not known whether Kir2.1 expression and contributions are altered in anti-inflammatory states. We tested the hypothesis that Kir2.1 contributes to Ca^2+^ entry, proliferation and migration of rat microglia. Kir2.1 (*KCNJ2*) transcript expression, current amplitude, and proliferation were comparable in unstimulated microglia and following alternative activation (IL-4 stimulated) and acquired deactivation (IL-10 stimulated). To examine functional roles of Kir2.1 in microglia, we first determined that ML133 was more effective than the commonly used blocker, Ba^2+^; i.e., ML133 was potent (IC_50_ = 3.5 μM) and voltage independent. Both blockers slightly increased proliferation in unstimulated or IL-4 (but not IL-10)-stimulated microglia. Stimulation with IL-4 or IL-10 increased migration and ATP-induced chemotaxis, and blocking Kir2.1 greatly reduced both but ML133 was more effective. In all three activation states, blocking Kir2.1 with ML133 dramatically reduced Ca^2+^ influx through Ca^2+^-release-activated Ca^2+^ (CRAC) channels. Thus, Kir2.1 channel activity is necessary for microglial Ca^2+^ signaling and migration under resting and anti-inflammatory states but the channel weakly inhibits proliferation.

## Introduction

Members of the Kir2 inward-rectifier K^+^ channel family (which includes Kir2.1) are expressed in both excitable and non-excitable cells, where their primary function is to maintain a hyperpolarized membrane potential (Lu, 2004; Hibino et al., 2010). Kir2.1 currents have often been reported in cultured rodent microglia (reviewed in Kettenmann et al., 1993, 2011; Eder, 2005) in both unstimulated (often called “resting”) and classical-activated (pro-inflammatory) states (Nörenberg et al., 1992, 1994; Visentin et al., 1995; Schlichter et al., 1996; Chung et al., 1999; Draheim et al., 1999; Prinz et al., 1999; Franchini et al., 2004; Newell and Schlichter, 2005; Moussaud et al., 2009). The current has also been recorded in microglia in brain slices (Brockhaus et al., 1993; Boucsein et al., 2000, 2003; Lyons et al., 2000; Schilling and Eder, 2007, 2015). After CNS damage, microglia can also enter anti-inflammatory states that help resolve classical activation and promote repair (reviewed in Hanisch and Kettenmann, 2007; Colton, 2009; Czeh et al., 2011). However, it is not known if Kir2.1 is expressed in microglia in these states; i.e., following “alternative” activation (evoked by interleukin-4) or “acquired deactivation” (evoked by IL-10).

After CNS injury, the population of microglia at damage sites will depend on both proliferation and migration; thus, it is important to compare these functions in pro- and anti-inflammatory states. It is well known that cell proliferation and migration are Ca^2+^-dependent processes. Our early study showed that when rat microglia were exposed to colony-stimulating factor-1 (CSF-1) to increase proliferation; this was reduced by Ba^2+^ (5–10 mM) (Schlichter et al., 1996) but the microglial activation state was not determined. Another study showed that Ba^2+^ block of Kir2.1 reduced ATP-induced Ca^2+^ entry in rat microglia by prolonging membrane depolarization (Franchini et al., 2004). While suggestive of a link between Kir2.1, proliferation and Ca^2+^ signaling, previous studies have not addressed whether the microglial activation state affects Kir2.1 contributions.

We recently found that stimulation of rat microglia with IL-4 or IL-10 increases their migration, ATP-induced chemotaxis and invasion through extracellular matrix (ECM) while classical activation (induced by LPS) reduces these functions (Lively and Schlichter, 2013; Ferreira et al., 2014; Siddiqui et al., 2014). Both their migration and chemotaxis depend on Ca^2+^ influx through Ca^2+^-release activated Ca^2+^ (CRAC/Orai1) channels (Siddiqui et al., 2012; Ferreira and Schlichter, 2013), which are highly expressed in unstimulated rat microglia (Ohana et al., 2009; Siddiqui et al., 2012). However, CRAC-mediated Ca^2+^ entry has not been compared for microglia in alternative- or acquired-deactivation states. CRAC is activated by depleting intracellular Ca^2+^ stores and is strongly inward-rectifying at negative membrane potentials (reviewed in Derler et al., 2012; Shim et al., 2015); thus, Ca^2+^ influx through CRAC is enhanced with hyperpolarization. Kir2.1, like other classical Kir channels, is expected to maintain a negative membrane potential.

Based on these previous results, we hypothesized that Kir2.1 will contribute to CRAC-mediated Ca^2+^ entry in unstimulated, IL-4- and IL-10-stimulated rat microglia, and this will be reflected by its contributions to proliferation and migration. Real-time RT-PCR was used to monitor expression of Kir2.1 (encoded by the *KCNJ2* gene) and patch-clamp recordings were used to compare Kir2.1 currents. Fura-2 imaging was used to quantify the contribution of Kir2.1 to CRAC-mediated Ca^2+^ signaling. Then, after demonstrating the utility of the recently developed Kir2-family inhibitor, ML133, we used Ba^2+^ and ML133 to assess Kir2.1 contributions to proliferation, migration and chemotaxis.

## Methods

### Rat Microglia Culture and Activation

All procedures on rats were approved by the University Health Network Animal Care Committee, and adhered to guidelines from the Canadian Council on Animal Care. Pure microglial cultures were prepared from 1–2 day-old Sprague- Dawley rat pups (Charles River, St-Constant, PQ, Canada) as described in our many previous publications (and recently in Lively and Schlichter, 2013; Ferreira et al., 2014; Siddiqui et al., 2014; Wong and Schlichter, 2014). In brief, brain tissue (minus cerebellum and meninges) was mashed in cold Minimal Essential Medium (MEM; Invitrogen, Carlsbad, CA), strained, and centrifuged at 300× g for 10 min. After re-suspending the cells in MEM, they were seeded in 75 cm^2^ flasks containing 30 ml of MEM supplemented with 10% fetal bovine serum (FBS; Wisent St-Bruno, PQ, Canada) and 0.05 mg/ml gentamycin (Invitrogen) and incubated at 37°C with 5% CO_2_. After 48 h, the medium was changed to remove cellular debris and non-adherent cells. Five to six days later, microglial cells were harvested by shaking the flasks for 5 h on an orbital shaker at 65 rpm (37°C, 5% CO_2_). The supernatant containing non-adherent cells was collected and centrifuged (300× g, 10 min) to obtain a microglia-rich pellet, which was re-suspended in fresh MEM (with 2% FBS with 0.05 mg/ml gentamycin). Microglia were seeded onto UV-irradiated 15 mm glass coverslips (Fisher Scientific, Ottawa, ON, Canada) at different densities depending on the experiment, as noted below. Microglia were unstimulated or stimulated for 6 or 24 h with 20 ng/ml of rat recombinant IL-4 (to induce alternative activation) or 20 ng/ml or rat recombinant IL-10 (for acquired deactivation) (both from R&D Systems Inc., Minneapolis, MN).

### Other Chemicals

For patch-clamp recordings, Ba^2+^ or ML133 were used to block Kir2.1, and agitoxin-2 was used to block Kv1.3 channels. Stock solutions were prepared in DMSO for ML133 (Tocris Bioscience, MO) and in double distilled water for BaCl_2_ and agitoxin-2, to which 0.02% BSA was added (all from Sigma). To examine Ca^2+^ signaling, thapsigargin and BTP2 (both from EMD Millipore Calbiochem, San Diego, CA) were prepared in DMSO. Inhibitor solutions were diluted to a working final concentration of 0.01%, aliquoted and stored at −20°C until used.

### Quantitative Real-Time Reverse-Transcriptase Polymerase Chain Reaction (qRT-PCR)

Total RNA was extracted using TRIzol (Invitrogen) from unstimulated or stimulated microglia that had been seeded at 1–2 × 10^6^ cells/coverslip in 35 mm dishes. RNA was purified using RNeasy Mini Kit (QIAGEN, Mississauga, ON, Canada). Primers for *KCNJ2* (which encodes the Kir2.1 channel) and the housekeeping gene, *HPRT1*, were designed using “Primer3Tes”^1^ as follows. *KCNJ2*: forward (5′- ACCGCTACAGCATCGTCTCT -3′) and reverse (5′-CTGCACTGTTGTCGGGTATG -3′); HPRT1: forward (5′- CAGTACAGCCCCAAAATGGT -3′) and reverse (5′- CAAGGGCATATCCAACAACA -3′). RNA samples were reverse transcribed using SuperScriptII RNase reverse transcriptase, according to the manufacturer’s instructions (Invitrogen). cDNA was then amplified using an ABI PRISM 7700 Sequence Detection System (PEBiosystems, Foster City, CA), with the following protocol: 50°C for 2 min, 95°C for 10 min, 40 cycles at 95°C for 15 s and 60°C for 60 s, and three dissociation steps (95°C for 15 s, 60°C for 15 s, 95°C for 15 s). The threshold cycle (CT) for *KCNJ2* was normalized to that of *HPRT1*.

### Whole-Cell Patch-Clamp Recordings

Coverslips bearing unstimulated or stimulated microglia (7–8 × 10^4^ cells/coverslip), were mounted in a 300 μl volume perfusion chamber (Model RC-25, Warner Instruments, Hamden, CT). Bath (external) solutions, with or without channel blockers, were perfused into the chamber using a gravity-driven perfusion system flowing at ~1 ml/min. Recordings were made at room temperature. The standard bath solution consisted of (in mM): 125 NaCl, 5 KCl, 1 CaCl_2_, 1 MgCl_2_, 10 HEPES, 5 D-glucose (pH 7.4; 290–300 mOsm/kg H_2_O). For all recordings, 5 nM AgTx-2 was added to the bath solution to block Kv1.3 currents. Recording pipettes were filled with an intracellular solution containing: 40 KCl, 100 KAsp, 1 MgCl_2_, 10 HEPES, 2 MgATP (pH 7.2; 290–300 mOsm/kg H_2_O), with 0.5 CaCl_2_ and 1 EGTA to buffer internal free Ca^2+^ to ~120 nM. Patch pipettes (4–7 MΩ resistance) were made from thin wall borosilicate glass (WPI, Sarasota, FL) pulled on a Narishige puller (Narishige Scientific, Setagaya-Ku, Tokyo) and fire polished with a microforge (MF 900; Narishige). The junction potential (−12.6 mV) was calculated with the utility in pCLAMP ver 9 (Molecular Devices, Sunnyvale, CA). Data were acquired using an Axopatch 200A amplifier and filtered at 5 Hz with a Digidata 1322A board, and acquisition and analysis were performed using pCLAMP 10 software (all from Axon Instruments).

### Cell Morphology, Viability and Proliferation Assays

Microglia (6 × 10^4^ cells/coverslip) were unstimulated or stimulated for 24 h with a cytokine: IL-4 or IL-10. When a Kir2.1 inhibitor (Ba^2+^ or ML133) was used, it was added at the same time as the cytokine. To examine viability, microglia were incubated with propidium iodide (500 nM, Invitrogen) for 1 h (37°C, 5% CO_2_) before fixing with 4% paraformaldehyde (Electron Microscopy Sciences, Hatfield, PA) for 10 min at room temperature. Cells were permeabilized with 0.2% Triton X-100 for 5 min, and washed with PBS (3×, 5 min each), and stained with FITC-conjugated tomato lectin (TL; 1:500, 15 min), and the nuclear dye, 4′, 6-diamidino-2-phenylindole (DAPI; 1:3000, 5 min; Invitrogen). After washing (3×, 5 min each), coverslips were mounted on glass slides using Dako mounting medium (Dako, Glostrup, Denmark). Five random fields were imaged at 20× or 40× magnification using the deconvolution microscope (DECON; Carl Zeiss, Jena, Germany). Counts of dead microglia (cells double-labeled with PI and DAPI) were normalized to the total number of DAPI-positive cells in 5 fields of view for each treatment condition.

For proliferation, we used the CyQUANT NF assay (Invitrogen). Microglia were seeded at 4 × 10^4^ cells/well of a 96-well flat-bottom plate and cultured in MEM with 2% FBS for 1–2 days (37°C, 5% CO_2_). Then, they were stimulated for 24 h with IL-4 or IL-10, with or without a Kir2.1 channel inhibitor (Ba^2+^ or ML133). The dye-binding solution was added to the wells, incubated for 30 min (37°C, 5% CO_2_), and then the fluorescence intensity was measured using a multi-label plate counter (Victor^3^ 1420, Perkin Elmer, Woodbridge, ON, Canada), with excitation at 485 nm and emission at 535 nM. Readings were taken for 0.1 s at 3 mm from the bottom of the plate, in triplicate and averaged. For analysis, the readings with each Kir2.1 blocker were normalized to the untreated unstimulated (control) group.

### Transwell Migration Assay

Microglia were seeded at 3× 10^4^ cells/filter in 200 μl of MEM with 2% FBS in the upper well of a Transwell migration chamber (VWR, Mississauga, ON, Canada), as we recently described (Lively and Schlichter, 2013; Ferreira et al., 2014; Siddiqui et al., 2014). After 30 min, 300 μl of MEM with 2% FBS was added to the lower well, and microglia were left unstimulated or stimulated with 20 ng/ml of IL-4 or IL-10. When used, a channel inhibitor (Ba^2+^ or ML133) was added for a further 23 h (24 h total incubation period at 37°C, 5% CO_2_), to allow time for cell migration through the 8 μm diameter holes in the filter. For chemotaxis, ATP (300 μM) was added to the lower well. After incubation, the Transwell inserts were washed with PBS, fixed in 4% paraformaldehyde for 15 min, and washed in PBS (3×, 5 min each). A Q-tip was used to remove cells from the top of the filter. Cells that had migrated to the other side were counted after staining with 0.3% crystal violet in methanol (~1 min) and rinsing with PBS to remove excess stain. Cells were counted from 5 random fields at 40× magnification using an Olympus CK2 inverted microscope (Olympus, Tokyo, Japan) and normalized to random transmigration of the untreated unstimulated group.

### Intracellular Ca^2+^ Measurements

Unstimulated or stimulated microglia (7–8 × 10^4^ cells/coverslip), were incubated (40 min, room temperature) with 3.5 μg/ml Fura-2AM (Invitrogen) in standard bath solution containing 2 mM CaCl_2_. The coverslip was mounted in a perfusion chamber and washed to remove any residual external Fura-2. Measurements were acquired at room temperature using a Nikon Diaphot inverted microscope, Retiga-EX camera (Q-imaging, Burnaby, BC, Canada), DG-4 arc lamp with excitation wavelength changer (Sutter Instruments, Novato, CA), and Northern Eclipse image acquisition software (Empix Imaging, Missisauga, ON, Canada). Cells were exposed to 340 and 380 nm excitation wavelengths, with the excitation shutter closed between acquisitions to prevent photobleaching. Ratios (340/380) were obtained using a 505 nm dichroic mirror and a 510 nm emission filter. For nominally Ca^2+^-free solution, CaCl_2_ was omitted. EGTA was not added because we found that it can rapidly deplete Ca^2+^ from immune cells (Schlichter and Sakellaropoulos, 1994).

### Statistics

Whole-cell currents and Fura-2 signals were analyzed using Origin ver 9.0 (OriginLab, Northampton, MA). Dose response curve-fitting and all other data were analyzed using GraphPad ver 6.01 (GraphPad Software, San Diego). All graphical data are presented as mean ± standard error of the mean (SEM) for the *n* values indicated. The statistical significance of results was analyzed with a paired or unpaired Student’s *t*-test, or using a one- or two-way analysis of variance (ANOVA). Results were considered significant if *p* < 0.05.

## Results

### The Microglial Kir2.1 Current is Blocked by ML133

We isolated whole-cell Kir2.1 currents from unipolar rat microglia that had a distinct lamellum and a uropod. This morphology corresponds with a high migratory capacity (Siddiqui et al., 2012, 2014; Vincent et al., 2012; Lively and Schlichter, 2013; Ferreira et al., 2014). Before performing cell function assays, it was important to examine the efficacy of the two blockers (Ba^2+^, ML133) that we planned to use to test the role of Kir2.1 in microglia. ML133 is a recently identified small molecule inhibitor of the Kir2-family, and it blocks Kir2.1 heterologously expressed in HEK cells with an IC_50_ of 1.8 μM at 7.4 pH (less effective at lower pH; Wang et al., 2011). Very few studies have used ML133 (Wang et al., 2011; Masia et al., 2015), and it has not been reported for microglia.

**Figure 1:**
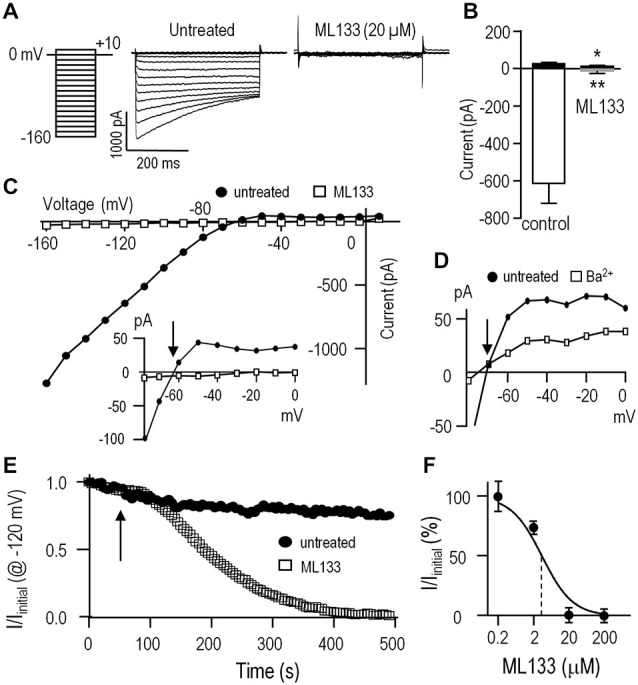
**The inward-rectifier Kir2.1 current in rat microglia is blocked by Ba^2+^ and ML133**. For all recordings, the standard bath solution contained 5 nM AgTx-2 to block the Kv1.3 current. Whole-cell currents were then recorded in response to the voltage protocol shown in panel **(A)**; i.e., test pulses between −160 and +10 mV in 10-mV increments from a holding potential of 0 mV. Note: in this, and all subsequent figures, the liquid junction potential was not corrected; thus, all voltages are 12.6 mV more negative than indicated. **(A)** Representative currents with and without the specific Kir2.x-family inhibitor, ML133 (20 μM). **(B)** Summary of amplitudes of inward current (at −120 mV) and outward current (at −50 mV) (mean ± SEM; *n* = 5 each) with and without 20 μM ML133. *p < 0.05, **p < 0.01; paired Student’s *t*-test. **(C)** Current-voltage (I-V) relations from the same cell as in A, before and after adding 20 μM ML133. *Inset*: I-V relations, plotted on an expanded Y-axis. Note the outward Kir2.1 current above the reversal potential, E_rev_ (arrow) before, but not after adding ML133. **(D)** I-V relations for a different cell before and after adding 100 μM Ba^2+^. Note the incomplete block of outward current. **(E)** Time course of block by 20 μM ML133, which was added to the bath at the time indicated by the arrow. Voltage steps to −120 mV from a holding potential of 0 mV were repeatedly applied at an interpulse interval of 5 s. **(F)** Dose-response curve for ML133 block of Kir2.1 current, normalized to the value without drug and presented as a semi-logarithmic function (mean ± SEM, *n* = 4–7 cells for each concentration). The IC_50_ (3.5 μM) is indicated by the vertical dashed line.

Kir2.1 presents as a rapidly activating, strongly inward-rectifying current, due to relief at negative potentials of block by internal Mg^2+^ and polyamines (reviewed in Hibino et al., 2010; Baronas and Kurata, 2014), and time-dependent block by external Na^+^ at very negative potentials (Kubo et al., 1993; Nörenberg et al., 1994). As illustrated in Figure 1A, the Kir current in rat microglia shows the hallmark rapid voltage-independent activation at negative potentials and time-dependent relaxation at very negative potentials. ML133 (at 20 μM) fully blocked the microglial Kir2.1 current at all voltages tested (Figures 1A–C). Importantly, ML133 blocked the small component of outward current just above the reversal potential (E_rev_; Figure 1C, inset). As expected for Kir2.1 current, there was strong inward rectification and E_rev_ was about −82 mV after junction potential correction, which is very close to the calculated Nernst potential with the bath and pipette solutions used (E_K_ = −85 mV). Although external Ba^2+^ is commonly used to block Kir2.1 currents in rodent microglia (Brockhaus et al., 1993; Nörenberg et al., 1994; Schlichter et al., 1996; Chung et al., 1999; Franchini et al., 2004), block is voltage dependent and decreases with membrane depolarization (Schlichter et al., 1996; Franchini et al., 2004). The outward Kir2.1 current in rat microglia was not well blocked by 100 μM external Ba^2+^ (Figure 1D). Caution is needed when using higher Ba^2+^concentrations in functional assays because millimolar Ba^2+^ also inhibit some voltage-dependent K^+^ channels (Armstrong and Taylor, 1980; Armstrong et al., 1982), and activates some SK channels (Cao and Houamed, 1999; Soh and Park, 2001). Therefore, in several of the following functional studies, we compared effects of ML133 with Ba^2+^.

We next examined the time dependence of block by bath-applied ML133 because it is known to act on an internal site (Wang et al., 2011) and should thus take time to enter the cell. As expected, full block by 20 μM ML133 required several minutes (Figure 1E). Note that in the absence of ML133, there was some time-dependent current rundown; i.e., to 79.4 ± 6.8% (*n* = 5) of the initial current at 5 min. This rundown is expected in whole-cell recordings due to loss of cytoplasmic components; e.g., lipid kinases that generate phosphatidylinositol 4, 5-bisphosphate, that are required to sustain Kir2.1 channel function (reviewed in Hilgemann et al., 2001; Fürst et al., 2014). Finally, a dose-response curve for ML133 block was constructed and yielded an IC_50_ of 3.5 μM (*n* = 4–7 cells for each point, Figure 1F). This value is comparable to the reported values in HEK cells (Wang et al., 2011) and murine neutrophils (Masia et al., 2015).

### Kir2.1 Expression and Current in Anti-Inflammatory Microglial Activation States

**Figure 2:**
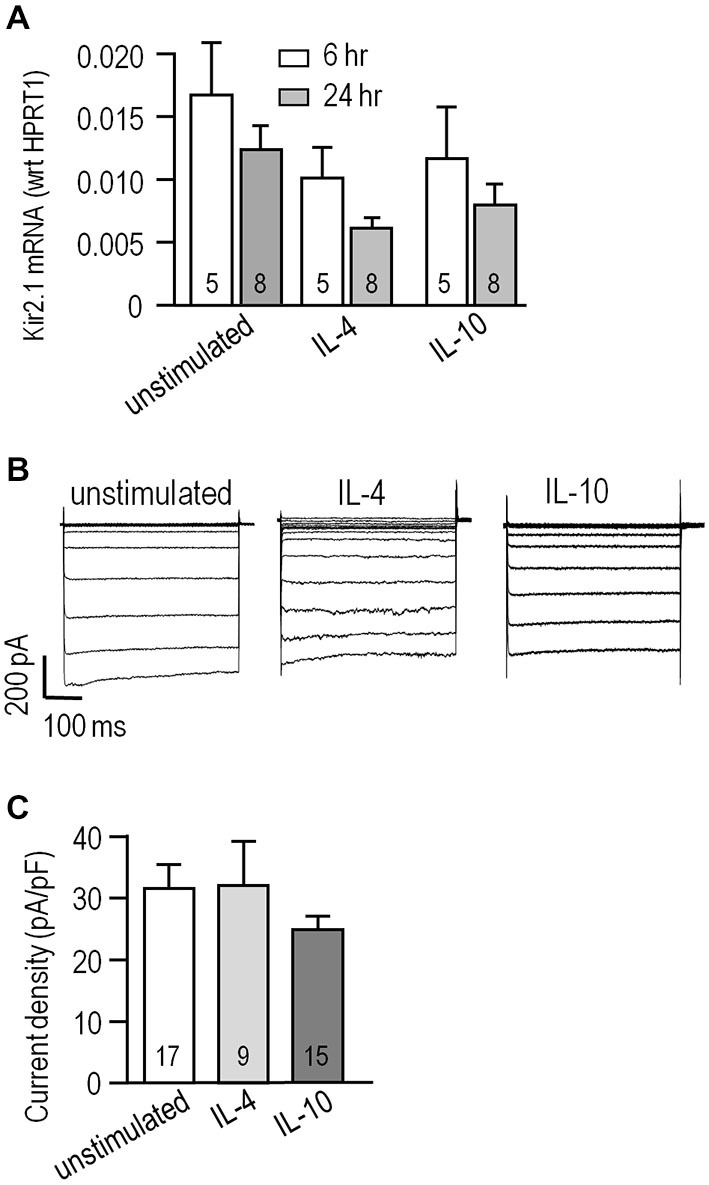
**Kir2.1 expression and current is unaffected by IL-4 or IL-10 stimulation. (A)** *KCNJ2* (Kir2.1) mRNA levels were monitored using real-time RT-PCR at 6 and 24 h from cells that were unstimulated or stimulated with 20 ng/ml IL-4 or IL-10. Channel expression was normalized to the housekeeping gene, *HPRT1*, and shown as mean ± SEM for the number of microglia cultures indicated on the bars. Comparisons based on a two-way analysis of variance (ANOVA) followed by Tukey’s *post hoc* test, indicate no significant differences. **(B)** Whole-cell Kir2.1 currents were recorded in response to voltage steps, as in Figure 1A. Representative recordings are shown for an unstimulated microglial cell and individual cells stimulated for 24 h with 20 ng/ml IL-4 or IL-10. **(C)** Summarized Kir2.1 current densities recorded at −120 mV, plotted as mean ± SEM for the number of cells indicated on each bar. A one-way ANOVA followed by a Bonferroni *post hoc* test indicates non-significant differences.

We previously found that alternative activation and acquired deactivation are elicited by 24 h stimulation of rat microglia with IL-4 or IL-10, respectively (Lively and Schlichter, 2013; Ferreira et al., 2014; Siddiqui et al., 2014). However, there are apparently no reports addressing expression of *KCNJ2* mRNA (which codes for Kir2.1) or Kir2.1 currents in microglia following stimulation with IL-4 or IL-10. Levels of *KCNJ2* mRNA did not significantly differ at 6 or 24 h after stimulation with IL-4 or IL-10 (Figure 2A). Nevertheless, it was important to compare the current amplitude in the different activation states because it can be affected by factors beyond mRNA expression; particularly, protein expression, trafficking to the surface membrane and post-translational modulation. We waited until 24 h after stimulation with IL-4 or IL-10 to allow time for such changes to occur. Representative currents are shown in Figure 2B. Figure 2C summarizes the current densities; i.e., the current measured at −120 mV was normalized to the cell capacitance, which is a measure of cell surface area. Current densities were not different between unstimulated cells and after IL-4 or IL-10. The capacitance did not statistically differ with treatments; i.e., it was 25 ± 2 pF (*n* = 18) for unstimulated microglia vs. 24 ± 2 pF (*n* = 17) for IL-10-stimulated cells; however, there was a trend toward a smaller cell size after IL-4 stimulation (19 ± 2 pF, *n* = 17; *p* = 0.14). We previously noted that IL-4 stimulated cells were generally smaller (Lively and Schlichter, 2013) but did not monitor their capacitance in that study.

### Kir2.1 Contributes to Microglial Proliferation, Migration and Chemotaxis

**Figure 3:**
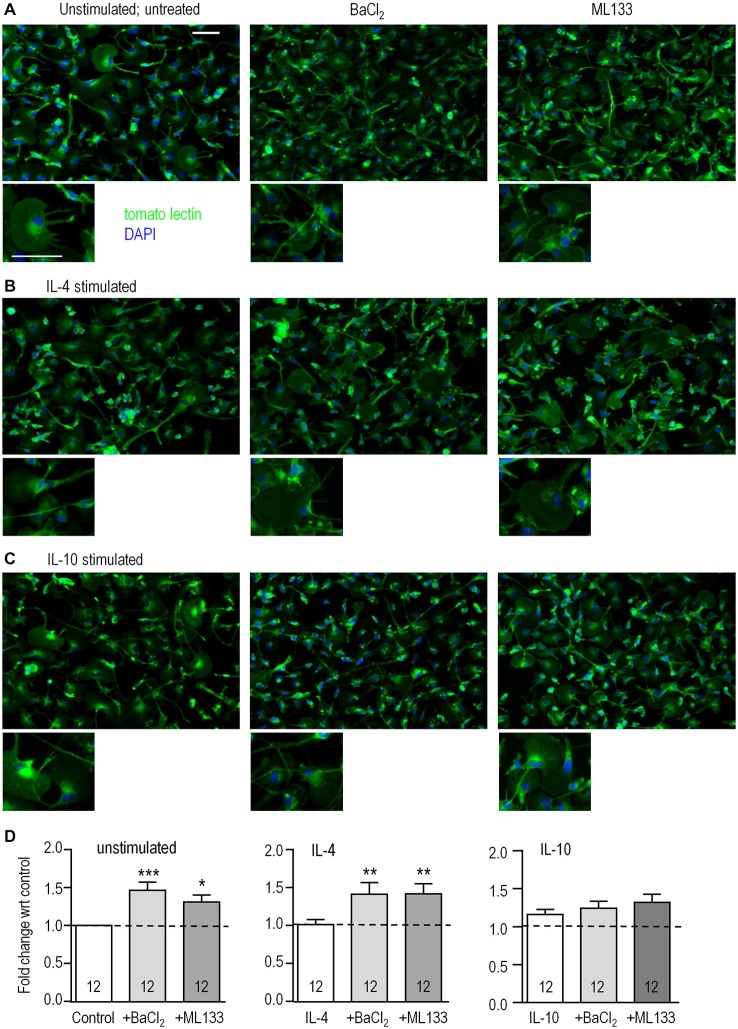
**Blocking Kir2.1 increases proliferation of unstimulated and IL-4-stimulated microglia**. Microglial cells were left unstimulated or stimulated with IL-4 or IL-10 for 24 h. When Kir2.1 blockers were used, they were added to the bath after the first hour and remained for the following 23 h. **(A–C)** Representative images of cells labeled with the microglia marker, tomato-lectin (green), and the nuclear dye, DAPI (blue): untreated (left panels), 1 mM Ba^2+^ (middle), 20 μM ML133 (right panels). [Note the purity of the microglial cultures, as all cells labeled with tomato lectin.] Scale bars are 50 μm and apply to all images. **(D)** For the CyQUANT proliferation assay, microglia were stimulated for 24 h with IL-4 or IL-10 in the presence or absence of Ba^2+^ or ML133 treatment. Results are expressed as fold change (mean ± SEM, *n* = 12 cultures per treatment) with respect to the untreated unstimulated control (dashed lines), and a one-way ANOVA with Bonferroni *post hoc* test was performed. *p < 0.05, **p < 0.01, ***p < 0.001.

We previously reported that rat microglia are migratory, with most cells having a unipolar morphology with a fan-shaped lamellum at the leading edge and a trailing uropod whether unstimulated or stimulated with IL-4 or IL-10 (Siddiqui et al., 2012, 2014; Vincent et al., 2012; Lively and Schlichter, 2013). This morphology was also prevalent in the present study (Figures 3A–C). However, when classically activated by LPS, they become non-migratory and dramatically change their morphology to nearly spherical (Lively and Schlichter, 2013; Siddiqui et al., 2014). Because both Kir2.1 blockers (Ba^2+^, ML133) greatly reduced migration (see below), we asked whether their morphology changed to that of LPS-stimulated cells. It did not; and blocking Kir2.1 did not obviously affect their unipolar morphology under any of the activation states examined (Figures 3A–C). Time-lapse imaging for 2 h (not shown) also showed that the blockers did not obviously affect their morphology. In addition, their viability, monitored with propidium-iodide, was >90% during the longest stimulation period (24 h) and was unaffected by activation state or treatment with ion channel blockers (not shown). However, from the images, we noted apparent differences in cell density after blocking Kir2.1, which we then quantified using the CyQuant assay in a microplate reader format. As we recently showed for IL-4 (Ferreira et al., 2014), proliferation was not affected by 24 h stimulation with IL-4 or IL-10 alone (Figure 3D). However, the Kir2.1 blockers significantly increased cell density (by 130–146%) of both unstimulated- and IL-4-stimulated microglia. Together, our results show that blocking Kir2.1 increased their proliferation without affecting viability. In contrast, this proliferative effect of blocking Kir2.1 was not seen in IL-10 stimulated microglia.

**Figure 4:**
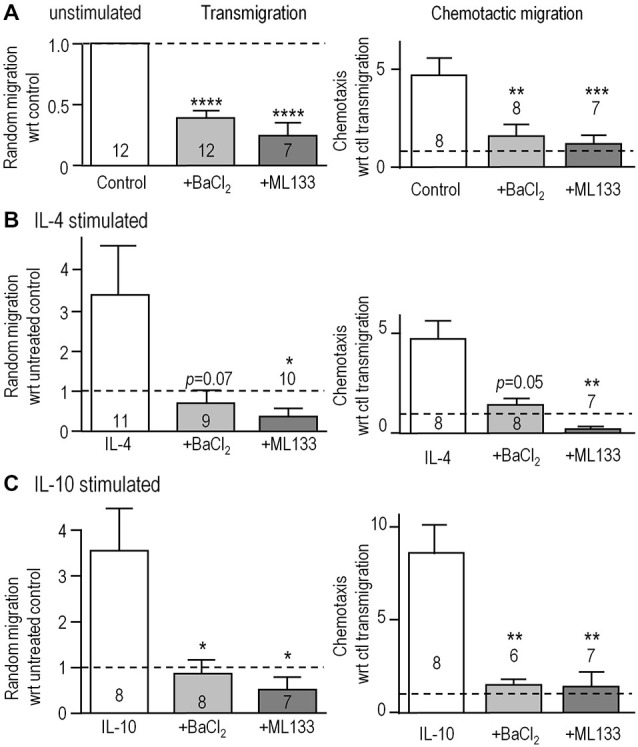
**Blocking Kir2.1 reduces migration of unstimulated, IL-4- and IL-10-stimulated microglia. (A–C)** Microglial cells were seeded on filters bearing 8 μm diameter pores in the upper wells of 3D Transwell™ chambers. We quantified random transmigration (left panels) and chemotactic migration induced by adding 300 μM ATP to the lower well (right panels). Microglia were unstimulated **(A)** or stimulated with IL-4 **(B)** or IL-10 **(C)** for 24 h, and when used, 1 mM Ba^2+^ or 20 μM ML133 was added after the first hour and for the remaining 23 h. All results are expressed as fold change with respect to random transmigration of untreated (control) unstimulated cells (i.e., 1.0 indicated by the dashed lines). Data are mean ± SEM for the number of microglial cultures indicated on each bar, and were analyzed using one-way ANOVA with Bonferroni *post hoc* test. *p < 0.05, **p < 0.01, ***p < 0.001, ****p < 0.0001.

We next asked if Kir2.1 is important for their migratory capacity. Transwell inserts were used to examine 3-dimensional migration in the absence (random transmigration) and presence of the chemoattractant, ATP (chemotactic migration). Consistent with our previous findings (Lively and Schlichter, 2013; Ferreira et al., 2014; Siddiqui et al., 2014), random transmigration was increased by stimulation with IL-4 (by 343%; *n* = 11) or IL-10 (344%; *n* = 8; Figure 4). Transmigration of unstimulated cells was reduced by blocking Kir2.1 with Ba^2+^ (by 61%) or ML133 (by 73%; Figure 4A). Similar, large reductions were seen in IL-4-stimulated cells (76% by Ba^2+^, 90% by ML133; Figure 4B) and IL-10-stimulated cells (77% by Ba^2+^, 85% by ML133; Figure 4C), and their migration was at or below the level of unstimulated cells (dotted lines). ATP-induced chemotactic migration was several-fold higher than random transmigration for all three activation states, and was dramatically reduced by treatment with Kir2.1 blockers; i.e., to about the same level as random transmigration of unstimulated cells (1.0 for the normalized data). It is important to note that these reductions are likely underestimated for unstimulated and IL-4-stimulated cells because the blockers increased proliferation (by 1.3–1.46 fold over 24 h; Figure 3D) and thus, increased the number of cells available to migrate during the assay.

### Blocking Kir2.1 Reduces Store-Operated Ca^2+^ Influx

**Figure 5:**
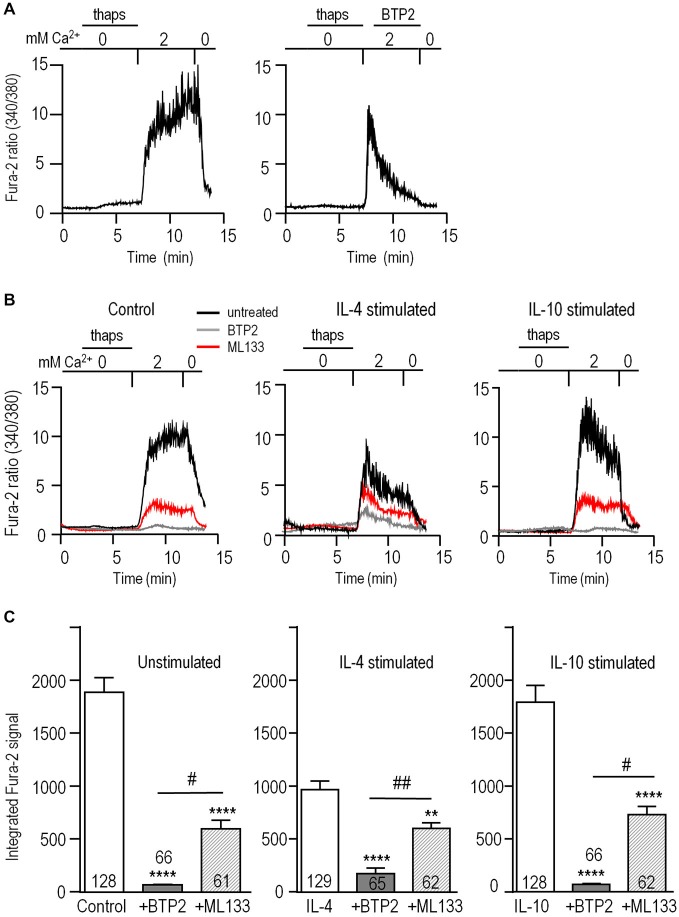
**Blocking Kir2.1 reduces CRAC-mediated Ca^2+^ influx**. Microglia were unstimulated or stimulated for 24 h with 20 ng/ml IL-4 or IL-10, and then loaded with the Ca^2+^-sensitive dye, Fura-2. The Ca^2+^ levels are reported as the ratio of the signal at 340 and 380 nm excitation wavelengths. **(A)** CRAC-mediated Ca^2+^ influx. As indicated by the horizontal bars above the traces, microglia were exposed for 5 min to Ca^2+^-free bath solution containing 1 μM thapsigargin (thaps) to deplete intracellular Ca^2+^ stores and activate CRAC. Then, Ca^2+^ influx was restored by perfusing in a solution containing 2 mM Ca^2+^. After 5 min, the dependence on external Ca^2+^ was confirmed by re-perfusing the bath with Ca^2+^-free solution. Right panel: A different cell, showing the response to the CRAC-channel blocker, 10 μM BTP2, added to the bath where indicated. **(B)** Reduction of the CRAC-mediated Ca^2+^ rise by blocking CRAC or Kir2.1. Representative recordings from unstimulated and IL-4- or IL-10 stimulated microglia, using the same protocol as in panel A. Ca^2+^ was monitored in separate coverslips of cells with or without 10 μM BTP2 or the Kir blocker, 20 μM ML133 present throughout the recording. **(C)** The area under each Fura-2 trace was integrated over the 5 min period of exposure to 2 mM Ca^2+^, and expressed in arbitrary units as mean ± SEM for the number of cells indicated on each bar. Three independent cell cultures were used to test each blocker (BTP2, ML133) and control cells were from the same 6 cell cultures. Results were analyzed using one-way ANOVA with a Bonferoni *post hoc* test. Statistical effects of each channel blocker within each activation state (stimulus) are indicated as **p < 0.01, ****p < 0.0001. Statistical differences between the blockers are indicated as ^#^*p* < 0.05, ^##^*p* < 0.01.

To examine whether Kir2.1 regulates CRAC-mediated Ca^2+^ influx in microglia, we isolated this component in Fura-2-loaded cells. As in our earlier study (Ohana et al., 2009), CRAC was activated by depleting Ca^2+^ stores using a 5-min treatment with 1 μM thapsigargin in a Ca^2+^-free bath solution. The baseline Fura-2 signal was low and stable, a large rise occurred when external Ca^2+^ was restored, and unstimulated microglia often displayed Ca^2+^ oscillations (Figure 5A). As expected for CRAC, the Ca^2+^ rise required influx and was rapidly eliminated by removing external Ca^2+^. Further evidence that the rise was CRAC-mediated was that it was eliminated by perfusing the CRAC blocker, 10 μM BTP2, into the bath.

Next, we assessed contributions of CRAC and Kir2.1 to Ca^2+^ influx in the different activation states (Figure 5B). To allow time for block by ML133 (Figure 1E) and BTP2 to develop (Figure 5A); each blocker was added to separate coverslips of cells for the duration of the experiment. Without channel blockers, the Ca^2+^ response of unstimulated microglia usually reached a plateau during the 5 min exposure to Ca^2+^; whereas, in IL-4- or IL-10-stimulated cells, there was usually a rapid rise followed by a spontaneous decline (Figure 5B). The Fura-2 signal, integrated over the 5-min exposure to external Ca^2+^, was ~50% lower in IL-4-stimulated microglia than in unstimulated- (*p* < 0.0001) or IL-10-stimulated cells (*p* < 0.0001; Figure 5C). The CRAC blocker, BTP2, reduced Ca^2+^ entry by 96% in both unstimulated and IL-10-stimulated cells (*p* < 0.0001), but was slightly less effective in IL-4-stimulated cells (reduced by 82%, *p* < 0.0001). As we predicted, blocking Kir2.1 with ML133 reduced Ca^2+^ entry in unstimulated cells (by ~68%, *p* < 0.001), and to a similar degree in IL-10-stimulated cells (~59%, *p* < 0.0001). After adding ML133 to IL-4-stimulated cells, the remaining signal was about the same as in unstimulated and stimulated cells. The percent reduction by ML133 was lower (~38%, *p* < 0.01), because the control IL-4 response was lower. Together, these results show that Ca^2+^ entry in microglia was mainly CRAC-mediated under all three activation states, and required Kir2.1 activity for maximal influx.

## Discussion

There are several salient findings in this study. (i) Expression of *KCNJ2* mRNA and Kir2.1 current were comparable in rat microglia that were unstimulated or had undergone alternative activation (with IL-4) or acquired deactivation (with IL-10). (ii) Proliferation was comparable in all three microglial activation states. It was slightly increased by blocking Kir2.1 in unstimulated and IL-4-stimulated microglia, but not after IL-10 stimulation. (iii) Migration and chemotaxis were increased by IL-4 and by IL-10, and were dramatically decreased by blocking Kir2.1 with Ba^2+^ or ML133, regardless of activation state. (iv) Blocking Kir2.1 with ML133 reduced Ca^2+^ influx through BTP2-sensitive, Ca^2+^-release-activated Ca^2+^ (CRAC) channels in all three activation states.

Reports of changes in Kir2.1 current with microglial activation have been inconsistent, possibly species dependent, and very little is known about anti-inflammatory states. For cultured murine microglia, several studies show that classical activation decreases Kir2.1 current; i.e., after exposure to lipopolysaccharide (LPS) or the pro-inflammatory cytokines, IFNγ or TNF-α (Draheim et al., 1999; Prinz et al., 1999; Boucsein et al., 2003). In contrast, effects on the current in rat microglia range from no change, to a small increase (LPS), to a large increase (IFNγ; Nörenberg et al., 1992; Visentin et al., 1995). For microglia in brain slices, Kir2.1 was observed in the murine corpus callosum and within the peri-infarct region after ischemia (middle cerebral artery occlusion) in rats (Brockhaus et al., 1993; Lyons et al., 2000). More recent studies have compared Kir2.1 currents in rodent microglia under different conditions: rat microglia in the facial nucleus before and after facial nerve axotomy, murine microglia from acute and cultured hippocampal slices, and murine microglia from young adult and aged animals (Boucsein et al., 2000; Schilling and Eder, 2007, 2015). The current was initially small in microglia from rat facial nucleus, acute murine hippocampal slices and young adult mice, and it increased in the denervated facial nucleus, in cultured slices, and in aged mice. However, microglial activation states were not determined. TGFβ, like IL-10, is considered a resolving cytokine (Suzumura et al., 1993; Pratt and McPherson, 1997), and in primary murine microglia or cell lines (BV-2, C8-B4) the current density was not affected by stimulation with TGFβ1 or TGFβ2 (Schilling et al., 2000; Moussaud et al., 2009). Here, we found that neither *KCNJ2* mRNA nor the Kir2.1 current differed under any of the activation states examined: unstimulated, IL-4- or IL-10-stimulated.

After comparing the Kir2.1 current in the different activation states, it was important to assess its contributions to microglial functions, for several reasons. The Kir2.1 current amplitude represents a snapshot in time, and changes in other currents could affect its relative contribution to the membrane potential and microglia functions over a longer period. In addition, Kir2.1 is subject to post-translational modulation (Hilgemann et al., 2001; Hibino et al., 2010; Fürst et al., 2014), which might differ in intact cells, with time, and with activation state. Results of functional studies using the common blocker, Ba^2+^, have been inconsistent; thus, it is important to consider its limitations, in particular that block is voltage dependent. The present study shows that Ba^2+^ is a poor blocker of outward Kir2.1 current in microglia. The reported bi-stable distribution of microglial membrane potentials (about −35 and −70 mV) (Nörenberg et al., 1994; Visentin et al., 1995; Boucsein et al., 2003) might explain why Ba^2+^ depolarized some rat microglial cells but did not affect others (Chung et al., 1999), and reduced the ATP-induced Ca^2+^ signal in some microglia but not others (Franchini et al., 2004). Here, we found that a higher Ba^2+^ concentration (1 mM) affected all the functions that were examined (proliferation, migration, chemotaxis) but relevant off-target effects have been reported. For instance, KCa2.3 and KCa3.1 channels are expressed in rat microglia (Kaushal et al., 2007; Schlichter et al., 2010; Siddiqui et al., 2012, 2014; Ferreira et al., 2014); 1 mM Ba^2+^ blocks cloned KCa3.1 channels (by 88%; Joiner et al., 1997), and sub-millimolar Ba^2+^ moderately activates cloned KCa2 and KCa3.1 channels (Cao and Houamed, 1999; Soh and Park, 2001). We previously showed that migration of rat microglia depends on KCa3.1 (Ferreira and Schlichter, 2013; Ferreira et al., 2014); thus, its block by Ba^2+^ could contribute to inhibition of migration and chemotaxis in the present study. Therefore, rather than relying only on Ba^2+^, we determined that the selective inhibitor, ML133 (Wang et al., 2011) blocked in a voltage-independent manner, with essentially complete block of both inward and outward Kir2.1 currents at 20 μM. Most importantly, ML133 greatly reduced CRAC-mediated Ca^2+^ entry and had the same effects as Ba^2+^ in increasing proliferation and reducing migration and chemotaxis. Each of these functional outcomes will be discussed in light of the literature.

The prevailing view is that Kir2.1 channel activity helps maintain a negative membrane potential in many cell types, and thereby regulates the driving force for ion fluxes, including Ca^2+^ influx. In rat microglia, Ca^2+^ can enter through several pathways, including Ca^2+^-release activated Ca^2+^ (CRAC/Orai1) and TRPM7 channels (Jiang et al., 2003; Ohana et al., 2009; Siddiqui et al., 2014), ionotropic purinergic receptors (Inoue, 2008), and reversed Na^+^/Ca^2+^ exchange (Newell et al., 2007). Here, we focussed on CRAC-mediated Ca^2+^ entry because the channel is highly Ca^2+^-selective and strongly inward-rectifying and thus, Ca^2+^ entry will be facilitated by hyperpolarization. CRAC accounted for essentially all of the store-operated Ca^2+^ entry (i.e., it was fully blocked by BTP2) in unstimulated and IL-10-stimulated microglia, and most of the Ca^2+^ entry in IL-4-stimulated cells. Blocking Kir2.1 with ML133 substantially reduced Ca^2+^ entry in all three microglial activation states, which provides the first evidence that Kir2.1 regulates microglia functions by promoting CRAC-mediated Ca^2+^ influx. Although beyond the scope of the present study, several observations regarding store-operated Ca^2+^ entry would be worth following up in future; i.e., differences in the shape of the Ca^2+^ signal in unstimulated vs. IL-4- or IL-10-stimulated microglia after Ca^2+^ was restored to the bath; and the lower Ca^2+^ response and BTP2-insensitive component in IL-4-stimulated cells.

Microglia must migrate and invade through ECM to reach damage sites, and this is stimulated by ATP release from damaged cells (Davalos et al., 2005; Färber and Kettenmann, 2006; Inoue, 2008; Tozaki-Saitoh et al., 2012). Cultured rat microglia are highly migratory (Siddiqui et al., 2012, 2014; Vincent et al., 2012; Ferreira and Schlichter, 2013; Lively and Schlichter, 2013; Ferreira et al., 2014), and both migration and invasion are increased in IL-4 and in IL-10 stimulated microglia (Lively and Schlichter, 2013; Ferreira et al., 2014; Siddiqui et al., 2014). Microglial migration is a Ca^2+^ dependent process involving CRAC channels (Siddiqui et al., 2012; Ferreira and Schlichter, 2013; Michaelis et al., 2015), as well as Ca^2+^-permeable TRPM7 channels in IL-4- and IL-10-stimulated cells (Siddiqui et al., 2014). In migrating rat microglia, CRAC/Orai1 is enriched in podosomes, which are tiny multi-molecular structures used for adhesion and ECM degradation during migration and invasion (Siddiqui et al., 2012; Vincent et al., 2012); and blocking CRAC reduced podosome expression, invasion and transmigration (Siddiqui et al., 2012). Consistent with the role of Kir2.1 in CRAC-mediated Ca^2+^ entry, we found that blocking Kir2.1 greatly reduced migration and ATP-induced chemotaxis in unstimulated, IL-4 or IL-10 stimulated microglia. Previous studies did not directly assess the role of Kir2.1 in migration. However, in murine microglia the Kir2.1 current and cell spreading were increased by activated Rac and decreased by activated Rho, which are small GTPases that regulate actin and facilitate cell migration (Muessel et al., 2013).

Neonatal rat microglia proliferate in culture (Schlichter et al., 1996). Consistent with our recent IL-4 study (Ferreira et al., 2014); proliferation was not altered by 24 h stimulation with IL-4 or IL-10. However, in unstimulated or IL-4-stimulated cells (but not after IL-10), proliferation was increased 40–46% by 1 mM Ba^2+^ and 30–46% by ML133. In our early study using the mitogen, CSF-1, 1 mM Ba^2+^ increased proliferation by 25%, while higher concentrations decreased it with an apparent IC_50_ of 1.5 mM (Schlichter et al., 1996). We now think that the inhibition at 5 and 10 mM Ba^2+^ was an off-target effect, possibly on KCa3.1, as noted above. Some studies have used targeted knockdown of Kir2.1 to avoid the problems associated with Ba^2+^. Consistent with our results using ML133 (and 1 mM Ba^2+^), proliferation of endothelial progenitor cells was increased after silencing Kir2.1 with siRNA or blocking it with Ba^2+^ (Jang et al., 2011) but the mechanism was not identified. The contribution of Kir2.1 to proliferation is somewhat controversial and might also depend on cell type. There was no effect or even reduced proliferation in Schwann cells, mesenchymal stem cells, smooth muscle cells, and fibroblasts after treatment with Ba^2+^ (10–500 μM) or dominant-negative suppression of Kir2.1 (Sobko et al., 1998; Karkanis et al., 2003; Zhang et al., 2012a,b; Qi et al., 2015). The mechanism by which Kir2.1 inhibition affects cell proliferation is not known. Numerous studies show that Ca^2+^ entry is important for cell proliferation (Capiod, 2011; Borowiec et al., 2014), and that cell cycle progression correlates with changes in K^+^ channel activity (reviewed in Pardo, 2004; Blackiston et al., 2009; Urrego et al., 2014), which is expected to affect the membrane potential. Unfortunately, detailed studies of membrane potential during the cell cycle are lacking for microglia and other cells. What is known is that the membrane K^+^ permeability is generally higher during the G1 phase and lower during other phases of the cell cycle, and this variation appears to be necessary because artificially sustained hyperpolarization blocks DNA synthesis (reviewed in Urrego et al., 2014). One possibility is that blocking Kir2.1 increased microglia proliferation by depolarizing the membrane, and shortening the G1 phase or increasing the rate of transition through S, G2 and M phases. If so, the lack of effect of blocking Kir2.1 on proliferation of IL-10-stimulated microglia might result from another K^+^ channel type providing the necessary high K^+^ permeability. Future studies will need to analyze K^+^ channel activity, membrane potential, and Ca^2+^ entry during the cell cycle, and determine whether they differ with microglial activation states.

In summary, the similar expression of Kir2.1 in unstimulated and anti-inflammatory activation states suggested that this channel is important for homeostatic functions of microglia. Proliferation and migration are two such functions, and there is evidence that these processes involve Ca^2+^ signaling and CRAC/Orai1 channels. The involvement of Kir2.1 in regulating membrane potential in other cell types, and the present results showing that Kir2.1 regulates CRAC-mediated Ca^2+^ entry in unstimulated and anti-inflammatory states, suggest a convergent mechanism. Conversely, classical-activated (LPS-stimulated) rat microglia are much less migratory than unstimulated or anti-inflammatory (IL-4- or IL-10-stimulated) cells (Lively and Schlichter, 2013; Ferreira et al., 2014; Siddiqui et al., 2014), and there is evidence that the Kir2.1 current is decreased in classical-activated mouse microglia (Draheim et al., 1999; Prinz et al., 1999; Boucsein et al., 2003). In future, the selective inhibitor, ML133, should prove useful for examining roles of Kir2.1 in classical-activated microglia and in other cell functions involved in CNS injury.

## Conflict of Interest Statement

The authors declare that the research was conducted in the absence of any commercial or financial relationships that could be construed as a potential conflict of interest.

## Footnotes

^1^http://flypush.imgen.bcm.tmc.edu/primer/primer3_www.cgi

## References

[B1] Armstrong C. M. Swenson R. P. Jr. Taylor S. R. (1982). Block of squid axon K channels by internally and externally applied barium ions. J. Gen. Physiol. 80, 663–682. 10.1085/jgp.80.5.663 6294220 PMC2228645

[B2] Armstrong C. M. Taylor S. R. (1980). Interaction of barium ions with potassium channels in squid giant axons. Biophys. J. 30, 473–488. 10.1016/s0006-3495(80)85108-3 6266531 PMC1328751

[B3] Baronas V. A. Kurata H. T. (2014). Inward rectifiers and their regulation by endogenous polyamines. Front. Physiol. 5:325. 10.3389/fphys.2014.00325 25221519 PMC4145359

[B4] Blackiston D. J. McLaughlin K. A. Levin M. (2009). Bioelectric controls of cell proliferation: ion channels, membrane voltage and the cell cycle. Cell Cycle 8, 3527–3536. 10.4161/cc.8.21.9888 19823012 PMC2862582

[B5] Borowiec A. S. Bidaux G. Pigat N. Goffin V. Bernichtein S. Capiod T. (2014). Calcium channels, external calcium concentration and cell proliferation. Eur. J. Pharmacol. 739, 19–25. 10.1016/j.ejphar.2013.10.072 24291106

[B6] Boucsein C. Kettenmann H. Nolte C. (2000). Electrophysiological properties of microglial cells in normal and pathologic rat brain slices. Eur. J. Neurosci. 12, 2049–2058. 10.1046/j.1460-9568.2000.00100.x 10886344

[B7] Boucsein C. Zacharias R. Farber K. Pavlovic S. Hanisch U. K. Kettenmann H. (2003). Purinergic receptors on microglial cells: functional expression in acute brain slices and modulation of microglial activation *in vitro*. Eur. J. Neurosci. 17, 2267–2276. 10.1046/j.1460-9568.2003.02663.x 12814360

[B8] Brockhaus J. Ilschner S. Banati R. B. Kettenmann H. (1993). Membrane properties of ameboid microglial cells in the corpus callosum slice from early postnatal mice. J. Neurosci. 13, 4412–4421. 8410196 10.1523/JNEUROSCI.13-10-04412.1993 PMC6576383

[B9] Cao Y. J. Houamed K. M. (1999). Activation of recombinant human SK4 channels by metal cations. FEBS Lett. 446, 137–141. 10.1016/s0014-5793(99)00194-5 10100630

[B10] Capiod T. (2011). Cell proliferation, calcium influx and calcium channels. Biochimie 93, 2075–2079. 10.1016/j.biochi.2011.07.015 21802482

[B11] Chung S. Jung W. Lee M. Y. (1999). Inward and outward rectifying potassium currents set membrane potentials in activated rat microglia. Neurosci. Lett. 262, 121–124. 10.1016/s0304-3940(99)00053-1 10203246

[B12] Colton C. A. (2009). Heterogeneity of microglial activation in the innate immune response in the brain. J. Neuroimmune Pharmacol. 4, 399–418. 10.1007/s11481-009-9164-4 19655259 PMC2773116

[B13] Czeh M. Gressens P. Kaindl A. M. (2011). The yin and yang of microglia. Dev. Neurosci. 33, 199–209. 10.1159/000328989 21757877

[B14] Davalos D. Grutzendler J. Yang G. Kim J. V. Zuo Y. Jung S.. (2005). ATP mediates rapid microglial response to local brain injury *in vivo*. Nat. Neurosci. 8, 752–758. 10.1038/nn1472 15895084

[B15] Derler I. Madl J. Schutz G. Romanin C. (2012). Structure, regulation and biophysics of I(CRAC), STIM/Orai1. Adv. Exp. Med. Biol. 740, 383–410. 10.1007/978-94-007-2888-2_16 22453951

[B16] Draheim H. J. Prinz M. Weber J. R. Weiser T. Kettenmann H. Hanisch U. K. (1999). Induction of potassium channels in mouse brain microglia: cells acquire responsiveness to pneumococcal cell wall components during late development. Neuroscience 89, 1379–1390. 10.1016/s0306-4522(98)00407-2 10362322

[B17] Eder C. (2005). Regulation of microglial behavior by ion channel activity. J. Neurosci. Res. 81, 314–321. 10.1002/jnr.20476 15929071

[B18] Färber K. Kettenmann H. (2006). Purinergic signaling and microglia. Pflugers Arch. 452, 615–621. 10.1007/s00424-006-0064-7 16791619

[B19] Ferreira R. Lively S. Schlichter L. C. (2014). IL-4 type 1 receptor signaling up-regulates KCNN4 expression and increases the KCa3.1 current and its contribution to migration of alternative-activated microglia. Front. Cell. Neurosci. 8:183. 10.3389/fncel.2014.00183 25071444 PMC4077126

[B20] Ferreira R. Schlichter L. C. (2013). Selective activation of KCa3.1 and CRAC channels by P2Y2 receptors promotes Ca^2+^ signaling, store refilling and migration of rat microglial cells. PLoS One 8:e62345. 10.1371/journal.pone.0062345 23620825 PMC3631179

[B21] Franchini L. Levi G. Visentin S. (2004). Inwardly rectifying K^+^ channels influence Ca^2+^ entry due to nucleotide receptor activation in microglia. Cell Calcium 35, 449–459 10.1016/j.ceca.2003.11.001 15003854

[B22] Fürst O. Mondou B. D’Avanzo N. (2014). Phosphoinositide regulation of inward rectifier potassium (Kir) channels. Front. Physiol. 4:404. 10.3389/fphys.2013.00404 24409153 PMC3884141

[B23] Hanisch U. K. Kettenmann H. (2007). Microglia: active sensor and versatile effector cells in the normal and pathologic brain. Nat. Neurosci. 10, 1387–1394. 10.1038/nn1997 17965659

[B24] Hibino H. Inanobe A. Furutani K. Murakami S. Findlay I. Kurachi Y. (2010). Inwardly rectifying potassium channels: their structure, function and physiological roles. Physiol. Rev. 90, 291–366. 10.1152/physrev.00021.2009 20086079

[B25] Hilgemann D. W. Feng S. Nasuhoglu C. (2001). The complex and intriguing lives of PIP2 with ion channels and transporters. Sci. STKE 2001:re19. 10.1126/scisignal.1112001re19 11734659

[B26] Inoue K. (2008). Purinergic systems in microglia. Cell. Mol. Life Sci. 65, 3074–3080. 10.1007/s00018-008-8210-3 18563292 PMC11131657

[B27] Jang S. S. Park J. Hur S. W. Hong Y. H. Hur J. Chae J. H.. (2011). Endothelial progenitor cells functionally express inward rectifier potassium channels. Am. J. Physiol. Cell Physiol. 301, C150–C161. 10.1152/ajpcell.00002.2010 21411724

[B28] Jiang X. Newell E. W. Schlichter L. C. (2003). Regulation of a TRPM7-like current in rat brain microglia. J. Biol. Chem. 278, 42867–42876. 10.1074/jbc.m304487200 12904301

[B29] Joiner W. J. Wang L. Y. Tang M. D. Kaczmarek L. K. (1997). hSK4, a member of a novel subfamily of calcium-activated potassium channels. Proc. Natl. Acad. Sci. U S A 94, 11013–11018. 10.1073/pnas.94.20.11013 9380751 PMC23566

[B30] Karkanis T. Li S. Pickering J. G. Sims S. M. (2003). Plasticity of KIR channels in human smooth muscle cells from internal thoracic artery. Am. J. Physiol. Heart Circ. Physiol. 284, H2325–H2334. 10.1152/ajpheart.00559.2002 12598232

[B31] Kaushal V. Koeberle P. D. Wang Y. Schlichter L. C. (2007). The Ca^2+^-activated K^+^ channel KCNN4/KCa3.1 contributes to microglia activation and nitric oxide-dependent neurodegeneration. J. Neurosci. 27, 234–244. 10.1523/jneurosci.3593-06.2007 17202491 PMC6672279

[B32] Kettenmann H. Banati R. Walz W. (1993). Electrophysiological behavior of microglia. Glia 7, 93–101. 10.1002/glia.440070115 7678582

[B33] Kettenmann H. Hanisch U. K. Noda M. Verkhratsky A. (2011). Physiology of microglia. Physiol. Rev. 91, 461–553. 10.1152/physrev.00011.2010 21527731

[B34] Kubo Y. Baldwin T. J. Jan Y. N. Jan L. Y. (1993). Primary structure and functional expression of a mouse inward rectifier potassium channel. Nature 362, 127–133. 10.1038/362127a0 7680768

[B35] Lively S. Schlichter L. C. (2013). The microglial activation state regulates migration and roles of matrix-dissolving enzymes for invasion. J. Neuroinflammation 10:75. 10.1186/1742-2094-10-75 23786632 PMC3693964

[B36] Lu Z. (2004). Mechanism of rectification in inward-rectifier K^+^ channels. Annu. Rev. Physiol. 66, 103–129. 10.1146/annurev.physiol.66.032102.150822 14977398

[B37] Lyons S. A. Pastor A. Ohlemeyer C. Kann O. Wiegand F. Prass K.. (2000). Distinct physiologic properties of microglia and blood-borne cells in rat brain slices after permanent middle cerebral artery occlusion. J. Cereb. Blood Flow Metab. 20, 1537–1549. 10.1097/00004647-200011000-00003 11083228

[B38] Masia R. Krause D. S. Yellen G. (2015). The inward rectifier potassium channel Kir2.1 is expressed in mouse neutrophils from bone marrow and liver. Am. J. Physiol. Cell Physiol. 308, C264–C276. 10.1152/ajpcell.00176.2014 25472961 PMC4312842

[B39] Michaelis M. Nieswandt B. Stegner D. Eilers J. Kraft R. (2015). STIM1, STIM2 and Orai1 regulate store-operated calcium entry and purinergic activation of microglia. Glia 63, 652–663. 10.1002/glia.22775 25471906

[B40] Moussaud S. Lamodière E. Savage C. Draheim H. J. (2009). Characterisation of K^+^ currents in the C8–B4 microglial cell line and their regulation by microglia activating stimuli. Cell Physiol. Biochem. 24, 141–152. 10.1159/000233240 19710528

[B41] Muessel M. J. Harry G. J. Armstrong D. L. Storey N. M. (2013). SDF-1α and LPA modulate microglia potassium channels through rho gtpases to regulate cell morphology. Glia 61, 1620–1628. 10.1002/glia.22543 23893870 PMC4783762

[B42] Newell E. W. Schlichter L. C. (2005). Integration of K^+^ and Cl^−^ currents regulate steady-state and dynamic membrane potentials in cultured rat microglia. J. Physiol. 567, 869–890. 10.1113/jphysiol.2005.092056 16020460 PMC1474215

[B43] Newell E. W. Stanley E. F. Schlichter L. C. (2007). Reversed Na^+^/Ca^2+^ exchange contributes to Ca^2+^ influx and respiratory burst in microglia. Channels (Austin) 1, 366–376. 10.4161/chan.5391 18690036

[B44] Nörenberg W. Gebicke-Haerter P. J. Illes P. (1992). Inflammatory stimuli induce a new K^+^ outward current in cultured rat microglia. Neurosci. Lett. 147, 171–174. 10.1016/0304-3940(92)90587-w 1491802

[B45] Nörenberg W. Gebicke-Haerter P. J. Illes P. (1994). Voltage-dependent potassium channels in activated rat microglia. J. Physiol. 475, 15–32. 10.1113/jphysiol.1994.sp020046 7514664 PMC1160352

[B46] Ohana L. Newell E. W. Stanley E. F. Schlichter L. C. (2009). The Ca^2+^ release-activated Ca^2+^ current (I(CRAC)) mediates store-operated Ca^2+^ entry in rat microglia. Channels (Austin) 3, 129–139. 10.4161/chan.3.2.8609 19411837

[B47] Pardo L. A. (2004). Voltage-gated potassium channels in cell proliferation. Physiology (Bethesda) 19, 285–292. 10.1152/physiol.00011.2004 15381757

[B48] Pratt B. M. McPherson J. M. (1997). TGF-β in the central nervous system: potential roles in ischemic injury and neurodegenerative diseases. Cytokine Growth Factor Rev. 8, 267–292. 10.1016/s1359-6101(97)00018-x 9620642

[B49] Prinz M. Kann O. Draheim H. J. Schumann R. R. Kettenmann H. Weber J. R.. (1999). Microglial activation by components of gram-positive and -negative bacteria: distinct and common routes to the induction of ion channels and cytokines. J. Neuropathol. Exp. Neurol. 58, 1078–1089. 10.1097/00005072-199910000-00006 10515231

[B50] Qi X. Y. Huang H. Ordog B. Luo X. Naud P. Sun Y.. (2015). Fibroblast inward-rectifier potassium current upregulation in profibrillatory atrial remodeling. Circ. Res. 116, 836–845. 10.1161/CIRCRESAHA.116.305326 25608527

[B51] Schilling T. Eder C. (2007). Ion channel expression in resting and activated microglia of hippocampal slices from juvenile mice. Brain Res. 1186, 21–28. 10.1016/j.brainres.2007.10.027 18005942

[B52] Schilling T. Eder C. (2015). Microglial K^+^ channel expression in young adult and aged mice. Glia 63, 664–672. 10.1002/glia.22776 25472417 PMC4359010

[B53] Schilling T. Quandt F. N. Cherny V. V. Zhou W. Heinemann U. Decoursey T. E. (2000). Upregulation of Kv1.3 K^+^ channels in microglia deactivated by TGF-beta. Am. J. Physiol. Cell Physiol. 279, C1123–1134. 11003593 10.1152/ajpcell.2000.279.4.C1123

[B54] Schlichter L. C. Kaushal V. Moxon-Emre I. Sivagnanam V. Vincent C. (2010). The Ca^2+^ activated SK3 channel is expressed in microglia in the rat striatum and contributes to microglia-mediated neurotoxicity *in vitro*. J. Neuroinflammation 7:4. 10.1186/1742-2094-7-4 20074365 PMC2819255

[B70] Schlichter L. C. Sakellaropoulos G. (1994). Intracellular Ca^2+^ signaling induced by osmotic shock in human T lymphocytes. Exp. Cell Res. 215, 211–222. 10.1006/excr.1994.1334 7957671

[B55] Schlichter L. C. Sakellaropoulos G. Ballyk B. Pennefather P. S. Phipps D. J. (1996). Properties of K^+^ and Cl^−^ channels and their involvement in proliferation of rat microglial cells. Glia 17, 225–236. 10.1002/(sici)1098-1136(199607)17:3<225::aid-glia5>3.0.co;2-# 8840164

[B56] Shim A. H. Tirado-Lee L. Prakriya M. (2015). Structural and functional mechanisms of CRAC channel regulation. J. Mol. Biol. 427, 77–93. 10.1016/j.jmb.2014.09.021 25284754 PMC4459506

[B57] Siddiqui T. Lively S. Ferreira R. Wong R. Schlichter L. C. (2014). Expression and contributions of TRPM7 and KCa2.3/SK3 channels to the increased migration and invasion of microglia in anti-inflammatory activation states. PLoS One 9:e106087. 10.1371/journal.pone.0106087 25148577 PMC4141841

[B58] Siddiqui T. A. Lively S. Vincent C. Schlichter L. C. (2012). Regulation of podosome formation, microglial migration and invasion by Ca^2+^-signaling molecules expressed in podosomes. J. Neuroinflammation 9:250. 10.1186/1742-2094-9-250 23158496 PMC3551664

[B59] Sobko A. Peretz A. Shirihai O. Etkin S. Cherepanova V. Dagan D.. (1998). Heteromultimeric delayed-rectifier K^+^ channels in Schwann cells: developmental expression and role in cell proliferation. J. Neurosci. 18, 10398–10408. 9852577 10.1523/JNEUROSCI.18-24-10398.1998 PMC6793353

[B60] Soh H. Park C. S. (2001). Inwardly rectifying current-voltage relationship of small-conductance Ca^2+^-activated K^+^ channels rendered by intracellular divalent cation blockade. Biophys. J. 80, 2207–2215. 10.1016/s0006-3495(01)76193-0 11325723 PMC1301412

[B61] Suzumura A. Sawada M. Yamamoto H. Marunouchi T. (1993). Transforming growth factor-beta suppresses activation and proliferation of microglia *in vitro*. J. Immunol. 151, 2150–2158. 8345199

[B62] Tozaki-Saitoh H. Tsuda M. M. Inoue K. (2012). P2Y receptors in microglia and neuroinflammation. Wiley Interdiscip. Rev. Membr. Transp. Signal. 1, 493–501 10.1002/wmts.46

[B63] Urrego D. Tomczak A. P. Zahed F. Stühmer W. Pardo L. A. (2014). Potassium channels in cell cycle and cell proliferation. Philos. Trans. R. Soc. Lond. B Biol. Sci. 369:20130094. 10.1098/rstb.2013.0094 24493742 PMC3917348

[B64] Vincent C. Siddiqui T. A. Schlichter L. C. (2012). Podosomes in migrating microglia: components and matrix degradation. J. Neuroinflammation 9:190. 10.1186/1742-2094-9-190 22873355 PMC3423073

[B65] Visentin S. Agresti C. Patrizio M. Levi G. (1995). Ion channels in rat microglia and their different sensitivity to lipopolysaccharide and interferon-gamma. J. Neurosci. Res. 42, 439–451. 10.1002/jnr.490420402 8568930

[B66] Wang H. R. Wu M. Yu H. Long S. Stevens A. Engers D. W.. (2011). Selective inhibition of the K(ir)2 family of inward rectifier potassium channels by a small molecule probe: the discovery, SAR and pharmacological characterization of ML133. ACS Chem. Biol. 6, 845–856. 10.1021/cb200146a 21615117 PMC3177608

[B67] Wong R. Schlichter L. C. (2014). PKA reduces the rat and human KCa3.1 current, CaM binding and Ca^2+^ signaling, which requires Ser332/334 in the CaM-binding C terminus. J. Neurosci. 34, 13371–13383. 10.1523/JNEUROSCI.1008-14.2014 25274816 PMC6608312

[B68] Zhang J. Chan Y. C. Ho J. C. Siu C. W. Lian Q. Tse H. F. (2012a). Regulation of cell proliferation of human induced pluripotent stem cell-derived mesenchymal stem cells via ether-a-go-go 1 (hEAG1) potassium channel. Am. J. Physiol. Cell Physiol. 303, C115–C125. 10.1152/ajpcell.00326.2011 22357737

[B69] Zhang X. H. Zhang Y. Y. Sun H. Y. Jin M. W. Li G. R. (2012b). Functional ion channels and cell proliferation in 3T3–L1 preadipocytes. J. Cell. Physiol. 227, 1972–1979. 10.1002/jcp.22925 21732368

